# Role of subsurface ocean in decadal climate predictability over the South Atlantic

**DOI:** 10.1038/s41598-018-26899-z

**Published:** 2018-06-04

**Authors:** Yushi Morioka, Takeshi Doi, Andrea Storto, Simona Masina, Swadhin K. Behera

**Affiliations:** 1 Application Laboratory, JAMSTEC, Yokohama, Japan; 2 Fondazione Centro Euro-Mediterraneo sui Cambiamenti Climatici (CMCC), Bologna, Italy; 3 Istituto Nazionale di Geofisica e Vulcanologia (INGV), Sezione di Bologna, Bologna, Italy

## Abstract

Decadal climate predictability in the South Atlantic is explored by performing reforecast experiments using a coupled general circulation model with two initialization schemes; one is assimilated with observed sea surface temperature (SST) only, and the other is additionally assimilated with observed subsurface ocean temperature and salinity. The South Atlantic is known to undergo decadal variability exhibiting a meridional dipole of SST anomalies through variations in the subtropical high and ocean heat transport. Decadal reforecast experiments in which only the model SST is initialized with the observation do not predict well the observed decadal SST variability in the South Atlantic, while the other experiments in which the model SST and subsurface ocean are initialized with the observation skillfully predict the observed decadal SST variability, particularly in the Southeast Atlantic. In-depth analysis of upper-ocean heat content reveals that a significant improvement of zonal heat transport in the Southeast Atlantic leads to skillful prediction of decadal SST variability there. These results demonstrate potential roles of subsurface ocean assimilation in the skillful prediction of decadal climate variability over the South Atlantic.

## Introduction

Decadal climate variability is one of key research issues for regional societies to mitigate its related damages and build long-term adaptation plans. Low-frequency variability of sea surface temperature (SST), sea ice and external forcings such as greenhouse gases and aerosols are important factors for generation of the decadal climate variability. As a key driver of the decadal climate variability, many studies have elaborated on understanding and predicting the decadal SST variability in the tropics^1–4^, the North Pacific^5,6^ and the North Atlantic^7–9^ where long-term observation data are available. On the other hand, significant advances in climate modeling and global ocean observation networks such as Argo floats are now bringing more attention to understanding and predicting the decadal SST variability in the data-sparse Southern Hemisphere.

The decadal climate variability in the South Atlantic was first identified by seminal studies^10,11^. A singular value decomposition (SVD) analysis of observation data in the late 20th century revealed that low-frequency fluctuations of SST and sea level pressure (SLP) with 14–16 year period exist in the South Atlantic and a north-south dipole structure of SST anomalies tends to appear accompanied with subtropical high variations. Performing long-term simulation with a coupled general circulation model (CGCM), a subsequent study^12^ suggested that the South Atlantic may exhibit multi-decadal variability with 25–30 year period as well. This involves variations in southward extension of the subtropical high, which results in modulating meridional heat transport and generating SST variability in the South Atlantic. The important roles of meridional heat transport and wind-induced mixing in the decadal SST variability of the South Atlantic were further reported in other climate modelling studies^13,14^.

Besides local air-sea interaction, remote influences from the tropical Pacific decadal variability such as the Interdecadal Pacific Oscillation (IPO)^15^ have been recently identified for the decadal SST variability in the South Atlantic^16^ through the well-known atmospheric teleconnection, the Pacific-South American modes (PSA)^17^. On the other hand, one recent study^18^ demonstrated in an eddy-resolving ocean general circulation model (OGCM) that the multi-decadal SST variability in the Atlantic Sector of the Southern Ocean can be generated as an intrinsic ocean variability mode through eddy-mean interaction and propagate eastward along the background eastward current. Since the decadal SST variability generated in the South Atlantic tends to migrate eastward and have potential influences on decadal climate variability in the southern Indian Ocean^14,19^, skillful prediction of the decadal SST variability in the South Atlantic is crucial for that of decadal climate variability in the southern Indian Ocean. Furthermore, downstream influences on southeast Australian climate through the atmospheric teleconnection have been recently reported^20^.

Although significant advances are made in understanding decadal climate variability in the South Atlantic, decadal climate predictability in the South Atlantic was described only in a part of global decadal climate prediction^3,21–23^ and not fully understood. Therefore, this study aims to investigate the decadal climate predictability in the South Atlantic by performing decadal reforecast experiments using a CGCM. Two types of ocean initialization schemes, providing initial conditions for the reforecast experiments, are adopted here to examine potential roles of surface and subsurface oceans in the decadal variability. One is the SST initialization in which the model SST is strongly nudged to the observed SST using the surface heat flux (see Methodology). The SST-initialization has been widely utilized for seasonal climate prediction^24–28^ and decadal climate prediction (e.g. North Atlantic^7,29^). The other is the initialization of subsurface ocean temperature and salinity using three dimensional variational (3DVAR) data assimilation^30,31^. By adopting the 3DVAR initialization scheme, Doi *et al*.^32^ have successfully demonstrated a significant improvement in predicting interannual climate variability in the tropical Indian Ocean. In the following section, relative roles of surface and subsurface ocean initializations in the decadal climate predictions are examined.

## Results

### Decadal climate variability and predictability in the South Atlantic

**Figure 1:**
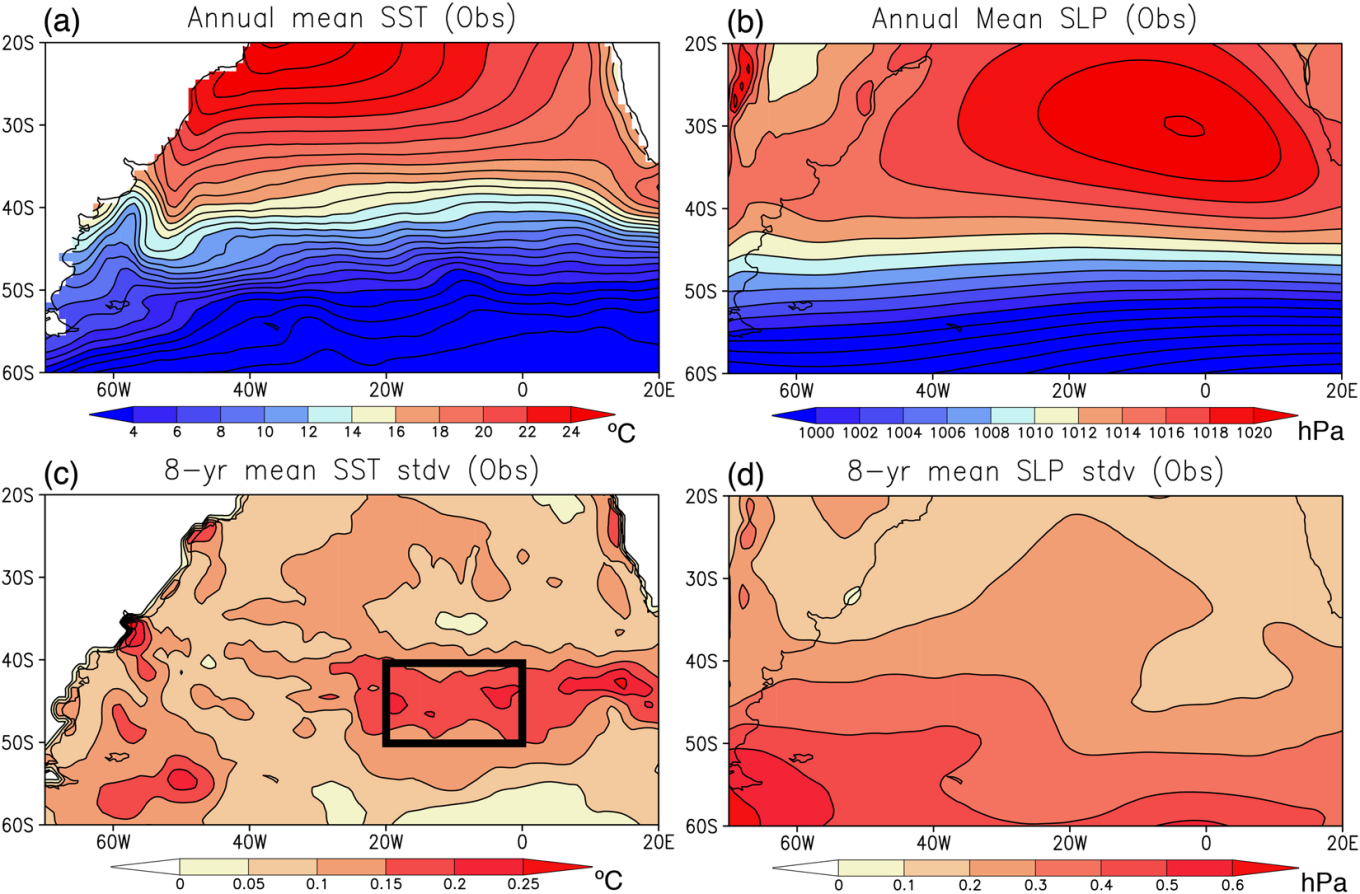
**(a)** Long-term mean SST (in °C) observed during 1982–2016. **(b)** Same as in **(a)**, but for the SLP (in hPa). **(c)** Standard deviation of 8-yr running mean detrended SST anomalies (in °C) from the observation. Black box indicates the Southeast Atlantic Ocean (SEAO; 20–0°W, 50–40°S) region of research interest where the low-frequency SST variability is large. **(d)** Same as in **(c)**, but for the standard deviation of the 8-yr running mean detrended SLP anomalies (in hPa). The maps were generated using Grid Analysis and Display System (GrADS) Version 2.1.a3 (http://cola.gmu.edu/grads/downloads.php).

Decadal climate variability is defined as low-frequency fluctuation from the mean state, so describing the mean state helps understand its decadal variability. Long-term mean SST during 1982–2016 (Fig. 1a) shows warm water intrusion by the southward Brazil Current and its confluence with cold water advection by the Malvinas Current along the South America. The confluence of the two western boundary currents leads to strong wind-driven eastward current, the South Atlantic Current at around 40°S. To the north of the South Atlantic Current (20–40°S), the subtropical high, the St. Helena High, dominates over the South Atlantic with its peak amplitude in the eastern side (Fig. 1b). In the Atlantic sector of the Southern Ocean (40–60°S), the low air pressure zonally extends over the cold SST, exhibiting prevalence of strong westerly winds.

The low-frequency SST variability, defined as standard deviation of the 8-year low-pass filtered SST anomalies using a simple running mean technique, is large along the Brazil Current and Malvinas Current (Fig. 1c). In analogy with other western boundary current regions (e.g. the North Pacific^33^), the fluctuations of these western boundary currents and their interaction with local atmosphere may be related to the low-frequency SST variability along the coast. Also, the northwestern South Atlantic shows slightly high variability, which may be related to decadal modulation of interannual variability, known as the South Atlantic subtropical dipole^34^ or the South Atlantic Ocean dipole^35,36^. The most noteworthy region in Fig. 1c, however, is the Southeast Atlantic where the SST has the largest variability with a zonal orientation. The SST variability could be related to the atmospheric variability, because the low-frequency SLP variability also exhibits a zonally elongated structure with larger variability to the south of the large SST variability (Fig. 1d).

**Figure 2:**
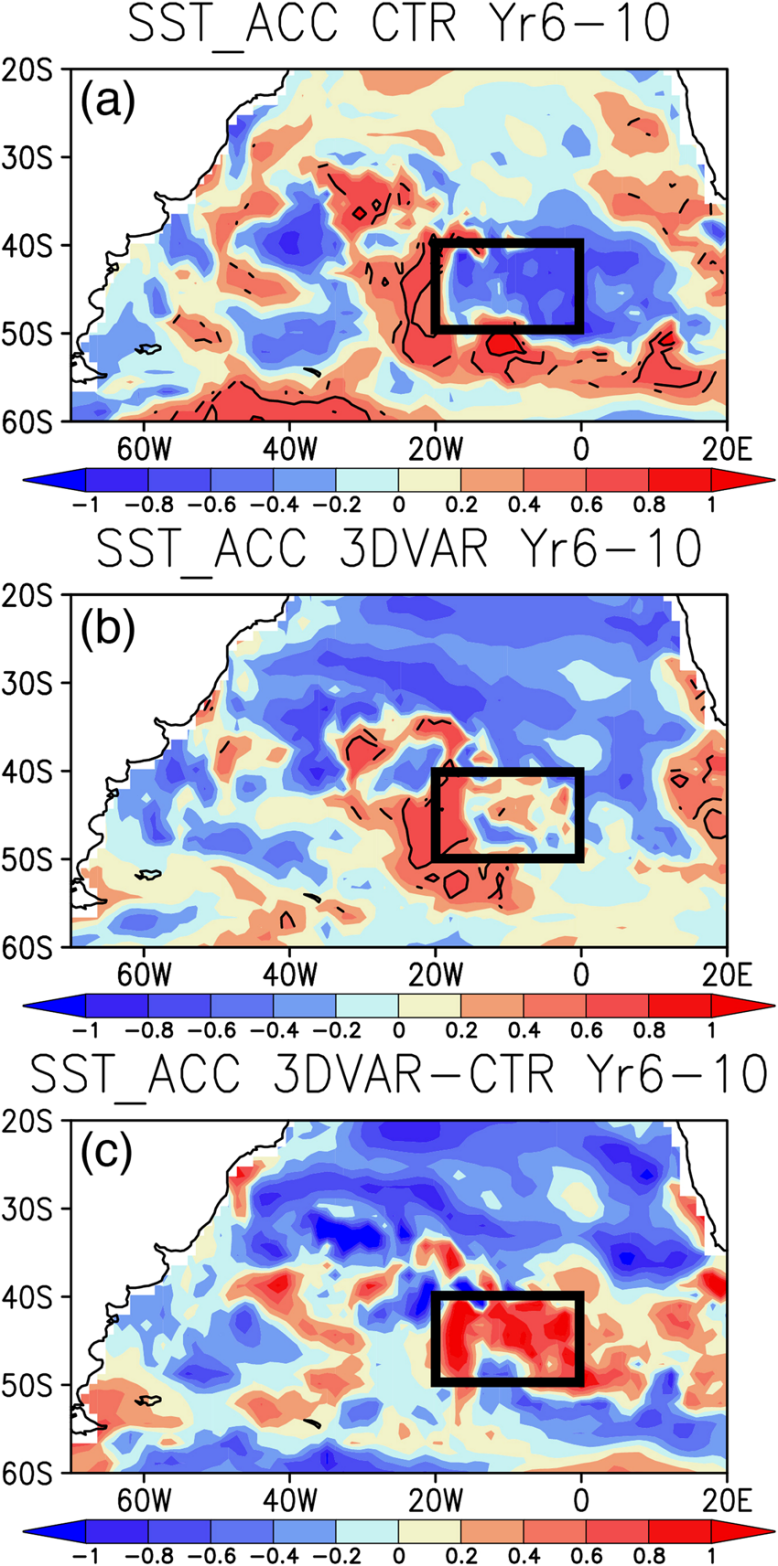
**(a)** Anomaly correlation coefficient (ACC) between the observed SST and the model SST predicated over 6–10 year leads in the CTR experiment. The positive ACC which is above the persistence (i.e. anomaly correlation between the observed SST and the SST over 6–10 year leads) and statistically significant at 90% confidence level of the two-tailed Student’s *t* test is shown by contour. Here we used the detrended SST anomalies for calculation of the ACC. For the model SST, a simple mean SST of 12 ensemble members was computed. Black boxes indicate the SEAO region defined in Fig. 1c. **(b)** Same as in **(a)**, but from the 3DVAR experiment. **(c)** Differences in the ACCs over 6–10 year leads between the CTR and 3DVAR experiments. The maps were generated using Grid Analysis and Display System (GrADS) Version 2.1.a3 (http://cola.gmu.edu/grads/downloads.php).

Decadal reforecast experiments exhibit some potentials in predicting the low-frequency SST variability. The ensemble mean of control (CTR) experiment initiated from Mar 1st of every year from 1982 to 2006 (i.e. 25 starting dates), in which only the model SST is initialized with the observed SST, shows moderately high skills above 0.6 anomaly correlation coefficient (ACC) in the southern South Atlantic, statistically significant over 6–10 year leads (Fig. 2a). Similarly as in the CTR experiment, the 3DVAR experiment, in which the model’s subsurface ocean temperature and salinity are additionally initialized using *in-situ* hydrographic profiles with the 3DVAR data assimilation scheme, presents moderately high skills in the southern South Atlantic, but the region with the moderately high ACC slightly shifts eastward along the 50–40°S compared to the CTR experiment. The differences in the ACCs between the two experiments indicate much improvement in the prediction skills of the Southeast Atlantic Ocean (SEAO; 20–0°W and 50–40°S) region for the 3DVAR experiment (Fig. 2c). Although the decadal SST predictability is spatially limited in the South Atlantic, the regions where the prediction skills show improvement correspond well with the regions where the decadal SST variability is pronounced (Fig. 1c), as suggested by Ding *et al*.^37^. In particular, the sea-surface observations show that decadal SST variability developed in the SEAO region migrates eastward along the background eastward current and contributes to the generation of decadal SST variability in the southern Indian Ocean^14^. So, it is crucial to understand and predict the genesis of this decadal SST variability in the SEAO region. Therefore, we have decided to select the SEAO region as the study area, as also suggested by previous studies^14,37^.

**Figure 3:**
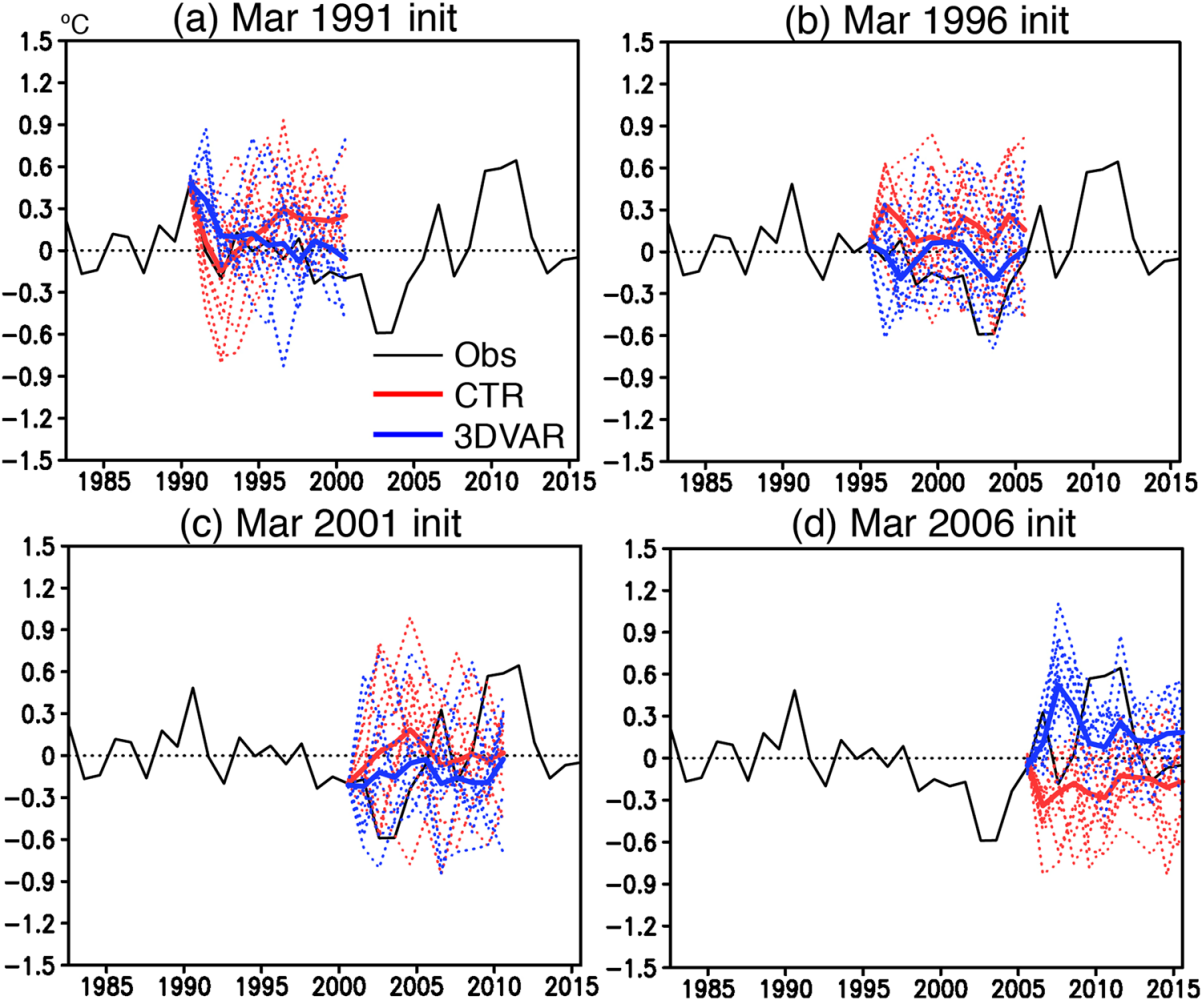
**(a)** Time series of detrended SST anomalies (in °C) averaged over the SEAO region (back box in Fig. 1c) from the observation (black line) and the CTR (red line) and 3DVAR (blue line) experiments initialized on Mar 1st 1991. For the CTR and 3DVAR experiments, the ensemble mean value (thick line) and each ensemble member (thin dotted line) are shown. (**b**–**d**) Same as in **(a)**, but for the case of the experiment initialized on Mar 1st 1996, 2001, and 2006, respectively. The maps were generated using Grid Analysis and Display System (GrADS) Version 2.1.a3 (http://cola.gmu.edu/grads/downloads.php).

The skillful SST prediction over 6–9 year leads in the 3DVAR experiment is confirmed in time series of SST anomalies averaged over the SEAO region. Since the subsurface ocean observation was very limited during 1980s (Fig. S1a,b), here we only show the time series after 1990s. In contrast to the CTR experiment, the 3DVAR experiment initialized in 1991 predicts a gradual decay from warm to cold phase during late 1990s (Fig. 3a). The cold phase in early 2000s is also predicted in the 3DVAR experiment initialized in 1996 (Fig. 3b), while the CTR experiment predicts warm phase. Although the 3DVAR experiment initialized in 2001 captures the cold phase in early 2000s, it does not predict a rapid shift from cold to warm phase during late 2000s (Fig. 3c). However, the 3DVAR experiment initialized in 2006 reasonably predicts the warm phase in late 2000s and early 2010s (Fig. 3d).

**Figure 4:**
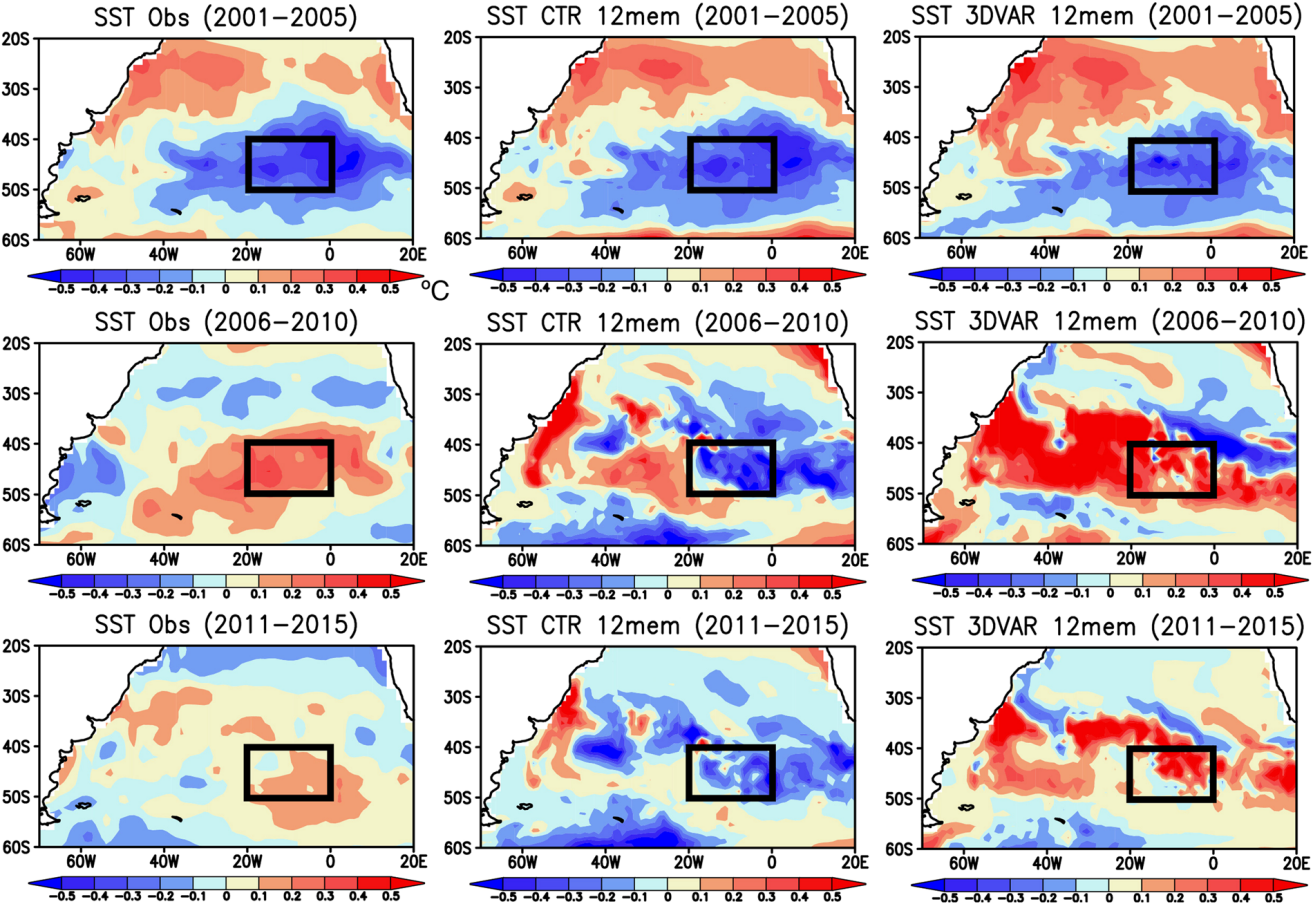
Spatial patterns of detrended SST anomalies (in °C) during (top) the initialization period of 2001–2005 and the prediction periods of (middle) 2006–2010 and (right) 2011–2015. From left to right, the SST anomalies from the observation, the CTR and 3DVAR experiments initialized on Mar 1st 2001 are shown. For the model SST, a simple mean SST of 12 ensemble members was computed. Black boxes correspond to the SEAO region defined in Fig. 1c. The maps were generated using Grid Analysis and Display System (GrADS) Version 2.1.a3 (http://cola.gmu.edu/grads/downloads.php).

A remarkable increase in number of subsurface observation (Fig. S1a,b) may contribute to more skillful prediction towards recent period in the 3DVAR experiment. This motivates us to further investigate the recent warm phase as a case study. During the warm phase of late 2000s and early 2010s, cold SST anomalies are initially observed in the SEAO region during 2001–2005, but they turn into warm SST anomalies during 2006–2010 with long persistence over 2011–2015 (Fig. 4). The CTR experiment initialized in 2006 does not capture the development of warm SST anomalies in the SEAO region, rather it predicts persistence of cold SST anomalies in late 2000s and early 2010s. On the other hand, the 3DVAR experiment initialized in 2006 successfully predicts the development of warm SST anomalies over the SEAO region in late 2000s and its long persistence in early 2010s.

**Figure 5:**
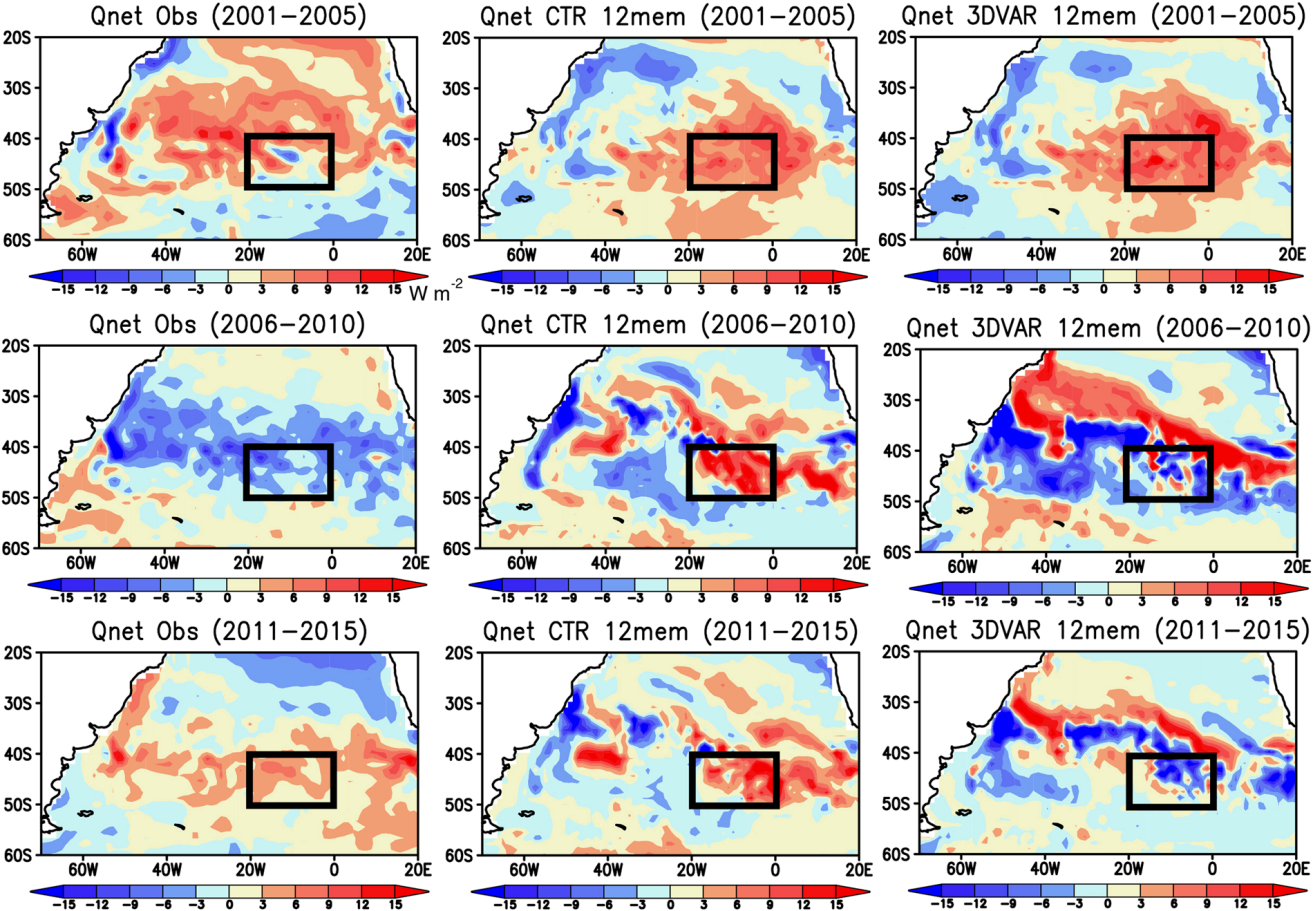
Same as in Fig. 4, but for the detrended surface heat flux anomalies (in W m^−2^). Positive values indicate that the atmosphere warms the ocean. The maps were generated using Grid Analysis and Display System (GrADS) Version 2.1.a3 (http://cola.gmu.edu/grads/downloads.php).

The skillful prediction of the warm SST anomalies in the 3DVAR experiment may not be linked to that of surface heat flux anomalies (Fig. 5). The surface heat flux anomalies observed during 2001–2005 show positive anomalies over the cold SST anomalies in the SEAO region, contributing to damping the cold SST anomalies. The counteracting effect of surface heat flux anomalies is also observed during 2006–2010. However, during 2011–2015, the positive surface heat flux anomalies act to keep the warm SST anomalies in the SEAO region. The inverse relationship between the SST and surface heat flux anomalies is more notable in both the CTR and 3DVAR experiments where the negative (positive) surface heat flux anomalies are generated over the warm (cold) SST anomalies in the South Atlantic.

The insignificant roles of atmospheric variations in the warm SST anomalies over the SEAO region are also suggested in the atmospheric circulation anomalies (Fig. S2). The SLP anomalies observed during 2001–2005 exhibit anticyclonic and cyclonic circulation anomalies to the west and east of the SEAO region, respectively. During the initialization period of 2001–2005, both the CTR and 3DVAR experiments show opposite signs of the observed circulation anomalies. In the atmospheric reanalysis, the positive SLP anomalies start to evolve in the SEAO region during 2006–2010, but turn into negative SLP anomalies during 2011–2015. However, the SLP anomalies predicted in both the CTR and 3DVAR experiments are negative during 2006–2010, opposite to the observed ones. On the other hand, both the CTR and 3DVAR experiments during 2011–2015 slightly reproduce the negative SLP anomalies in the SEAO region. The clear disagreement among the atmospheric reanalysis and the two experiments suggests that the successful prediction of the warm SST anomalies in the 3DVAR experiment may be more related to the low-frequency ocean variability induced by the subsurface ocean initialization than the atmospheric variability.

### Role of subsurface ocean in the South Atlantic decadal predictability

**Figure 6:**
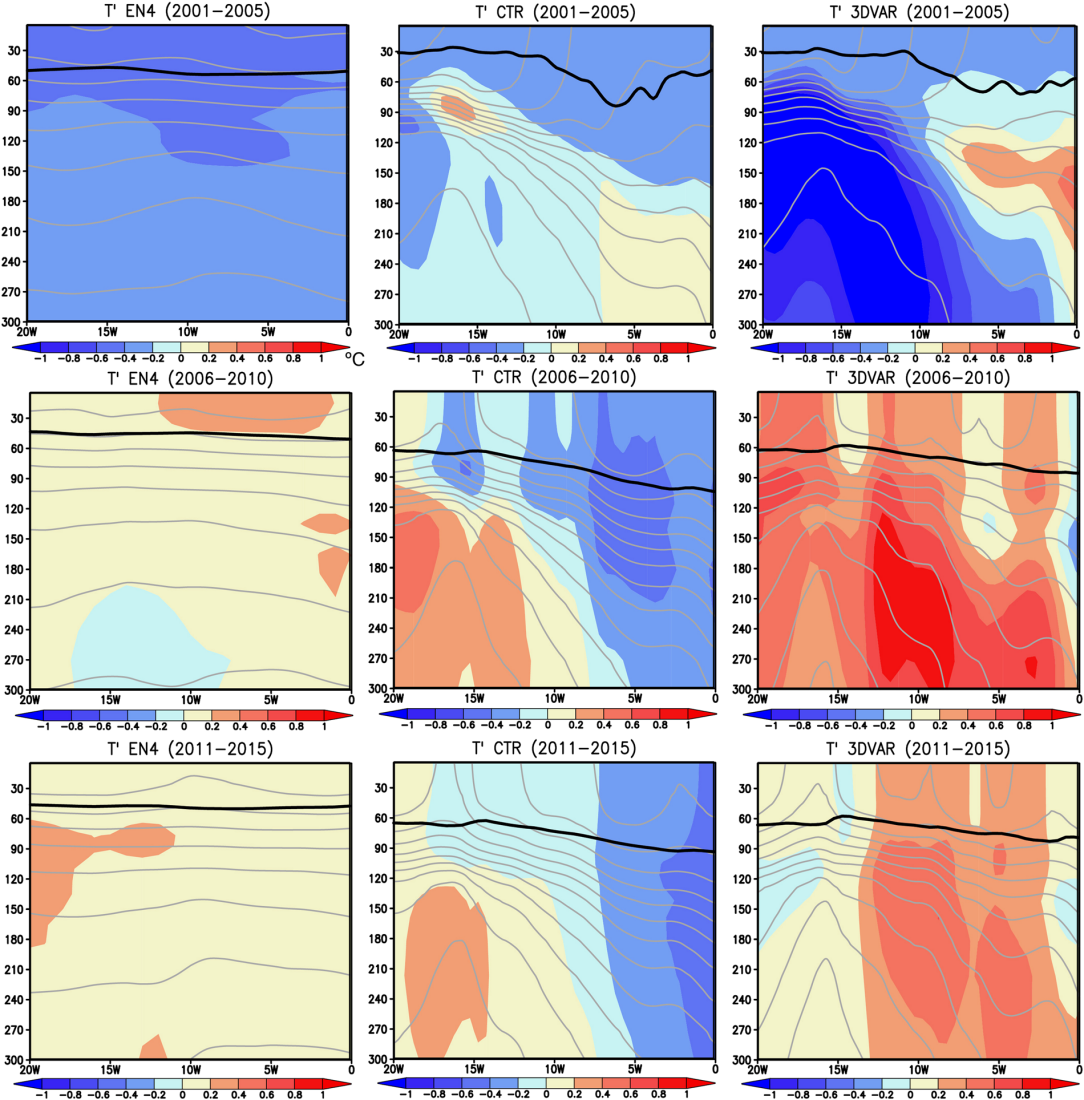
Same as in Fig. 4, but for the longitude-depth sections of detrended subsurface ocean temperature anomalies (in °C) averaged over 50–40°S of the Southeast Atlantic. Black and gray lines exhibit absolute mixed-layer depth and potential density (C. I. 0.1 Kg m^−3^), respectively. Here we defined the mixed-layer depth as the depth where the potential density increases by 0.125 Kg m^−3^ from the surface layer. The maps were generated using Grid Analysis and Display System (GrADS) Version 2.1.a3 (http://cola.gmu.edu/grads/downloads.php).

To investigate potential roles of subsurface ocean initialization in decadal predictability of the Southeast Atlantic, longitude-depth sections of ocean temperature anomalies, absolute mixed-layer depth and potential density averaged in 50–40°S are shown in Fig. 6. During the initialization period of 2001–2005, the observation shows that the cold temperature anomalies in the SEAO region (20–0°W) penetrate into deeper ocean below 300 m. The cold temperature anomalies in the subsurface ocean below 120 m are reproduced in both the CTR and 3DVAR experiments except for the subsurface warm temperature anomalies in the eastern part of the SEAO region, which is probably due to paucity of the *in-situ* observations (Fig. S1b). The amplitude of the cold temperature anomalies in the 3DVAR experiment is much larger than that in the CTR experiment. During the prediction period of 2006–2010, the observation exhibits development of warm temperature anomalies in the SEAO region. In contrast to the observation, the CTR experiment predicts cold temperature anomalies there. On the other hand, the 3DVAR experiment successfully predicts the development of warm temperature anomalies, in particular, in the eastern part of the SEAO region. The warm temperature anomalies are notably large below the mixed layer. Similar results are obtained for the prediction period of 2011–2015 when the observation shows warm temperature anomalies in the SEAO region. The deep structure of warm temperature anomalies is confirmed in the other ocean reanalysis product, although it shows cold temperature anomalies in the eastern part of the SEAO region (Fig. S3).

**Figure 7:**
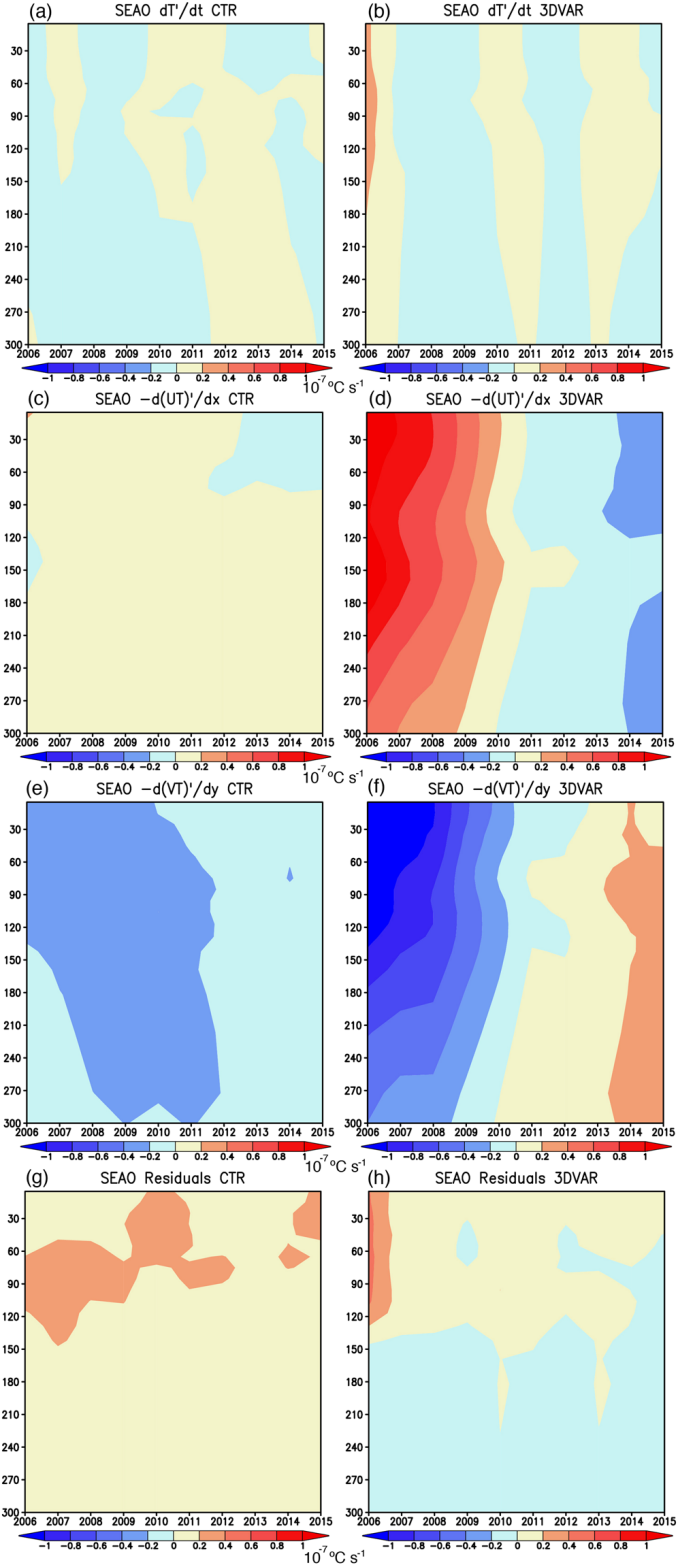
**(a**,**b)** Year-to-year variations of the detrended subsurface ocean temperature tendency anomalies (in 10^−7^ °C s^−1^) averaged over the SEAO region (black box in Fig. 1c) from the CTR and 3DVAR experiments, respectively. **(c**,**d)** Same as in **(a**,**b)**, but for the contributions from convergence anomalies of the horizontal heat transport (in 10^−7^ °C s^−1^) to the total temperature tendency anomalies. **(e**,**f)** Same as in **(c**,**d)**, but for the contribution from convergence anomalies of the meridional heat transport (in 10^−7^ °C s^−1^) to the total temperature tendency anomalies. **(g**,**h)** Same as in **(c**,**d)**, but for the contribution from the residual heat transport (in 10^−7^ °C s^−1^). The maps were generated using Grid Analysis and Display System (GrADS) Version 2.1.a3 (http://cola.gmu.edu/grads/downloads.php).

The impact of subsurface ocean initialization in the 3DVAR experiment is evaluated by performing heat budget analysis in the SEAO region (see Methodology). The temperature tendency anomalies in the 3DVAR experiment show larger positive values in 2006 than those in the CTR experiment (Fig. 7a,b). Among the three components contributing to the temperature tendency anomalies, it is the convergence anomalies of zonal heat transport that explain most of the positive temperature tendency anomalies in the 3DVAR experiment (Fig. 7d). The convergence anomalies of meridional heat transport act to dampen the anomalies in temperature tendency (Fig. 7f). There is secondary contribution from the residual component in the 3DVAR experiment (Fig. 7h), but it is vertically limited in the upper ocean above 120 m including the mixed layer (not shown) and the amplitude is smaller than the convergence anomalies of zonal heat transport (Fig. 7d). Since the surface heat flux anomalies in the 3DVAR experiment show negative values in the SEAO region during 2006–2015 (Fig. 5), ocean processes such as vertical advection and mixing could contribute to the positive anomalies in temperature tendency (Fig. 7h). Similar contribution from the convergence anomalies of zonal heat transport is confirmed in the other ocean reanalysis product, particularly during 2006–2011 (Fig. S4). The initialization of ocean heat transport may be crucial for development of warm temperature anomalies in the SEAO region after 2006.

**Figure 8:**
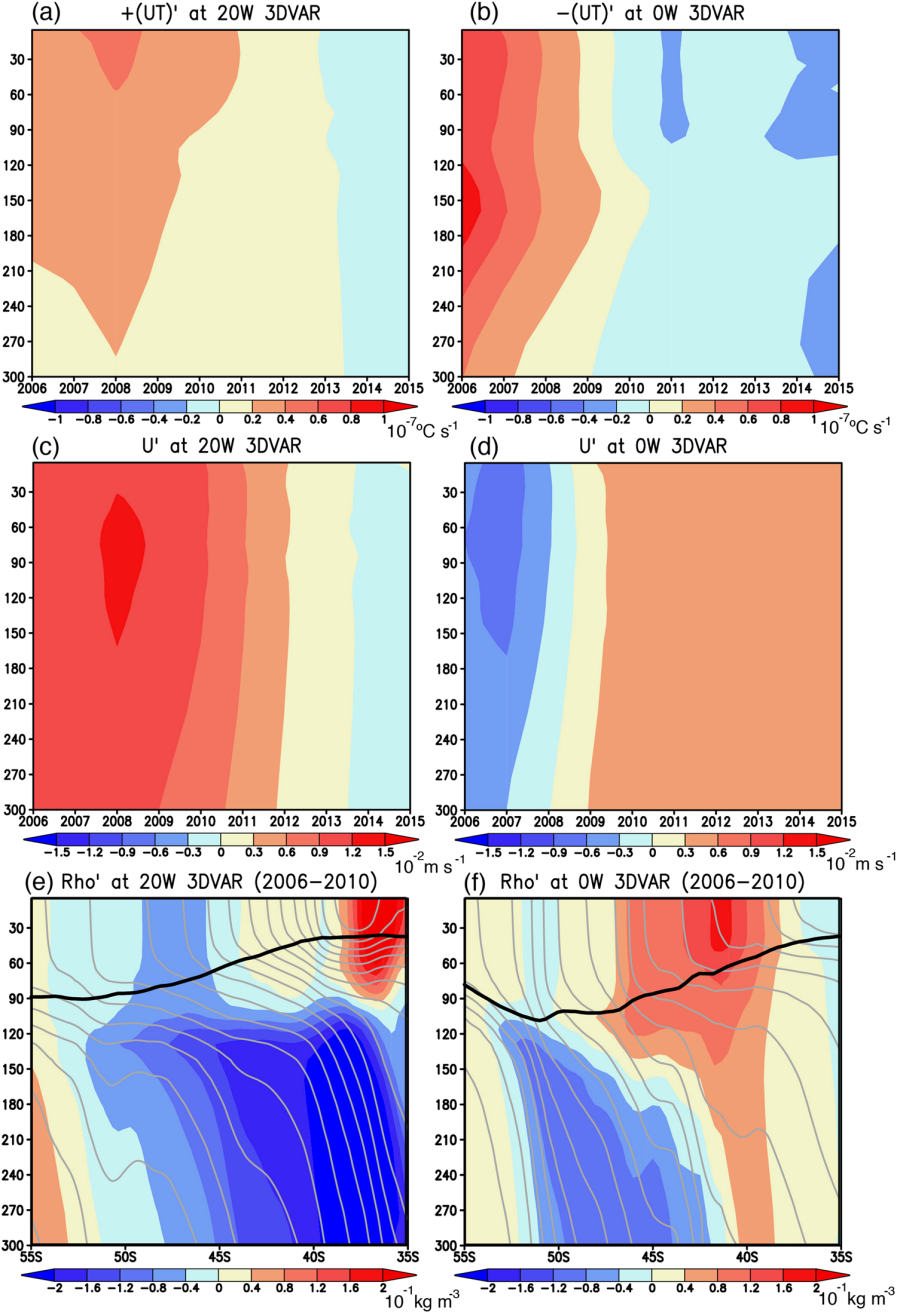
**(a)** Year-to-year variations of detrended horizontal heat transport anomalies (in 10^−7^ °C s^−1^) at the west boundary (20°W) of the SEAO region (black box in Fig. 1c) from the 3DVAR experiment. **(b)** Same as in **(a)**, but at the east boundary (0°W) of the SEAO region. For comparison with **(a)**, the values are multiplied with minus one. **(c**,**d)** Same as in **(a)**, but for the detrended zonal current anomalies (in 10^−2^ m s^−1^) at the west and east boundaries of the SEAO region, respectively. **(e**,**f)** Latitude-depth sections of detrended subsurface potential density anomalies (in 10^−1^ Kg m^−3^) at the west and east boundaries of the SEAO region, respectively. Black and gray lines exhibit absolute values of the mixed-layer depth and the potential density (C. I. 10^−1^ Kg m^−3^). The maps were generated using Grid Analysis and Display System (GrADS) Version 2.1.a3 (http://cola.gmu.edu/grads/downloads.php).

The convergence anomalies of zonal heat transport in the 3DVAR experiment were further decomposed into contributions from west (20°W) and east (0°W) boundaries of the SEAO region. Although slightly stronger at the east boundary, the zonal heat transport anomalies at both boundaries contribute to the positive convergence anomalies (Fig. 8a,b). This is mainly due to anomalous zonal convergence of mean warm temperature by zonal current anomalies (Fig. 8c,d), because anomalous zonal convergence of temperature anomalies (~0.5 °C, not shown) by mean eastward current (~0.05 m s^−1^) is estimated to be smaller than that of mean warm temperature (~10 °C) by zonal current anomalies (~10^–2^ m s^−1^). The latitude-depth section of potential density anomalies at the west boundary during 2006–2010 shows high and low values in the surface mixed-layer to the northern (45–35°S) and southern (55–45°S) parts of the SEAO region (Fig. 8e). The associated southward pressure gradient anomaly is geostrophically balanced by the Coriolis force exerted on the eastward current anomalies (Fig. 8c). On the other hand, at the east boundary, the high potential density anomalies in the surface mixed-layer shift southward (50–40°S) followed by the low potential density anomalies to the north (35°S, Fig. 8f). The associated northward pressure gradient anomalies may be linked to the westward current anomalies (Fig. 8d).

One may wonder if the convergence anomalies of the meridional heat transport during 2011–2015 also contribute to the positive temperature tendency anomalies in the 3DVAR experiment (Fig. 7b,f). Detailed analysis (Fig. S5a,b) reveals that the meridional heat transport anomalies at the north boundary (40°S) of the SEAO region contribute more than those at the south boundary (50°S). This is mainly attributed to anomalous meridional advection of mean warm temperature by anomalous southward current during 2011–2015 (Fig. S5c). The southward current anomalies seem to be related with the westward pressure gradient induced by the high and low potential density anomalies to the east (10°W–5°E) and west (25–10°W) of the SEAO region (Fig. S5d). The contribution from the anomalous meridional heat transport is also reported in previous results^12–14^.

## Discussion

An improvement of decadal climate prediction in the Southeast Atlantic is demonstrated by initializing the subsurface ocean in a CGCM with a 3DVAR data assimilation scheme. A better representation of zonal heat transport associated with the anomalous zonal current contributes to skillful prediction of warm SST anomalies in the Southeast Atlantic in late 2000s and early 2010s. The results show that both the observed and predicted surface heat flux anomalies tend to dampen the SST anomalies and the atmospheric anomalies do not seem to contribute much to generation of the warm SST anomalies in contrast to previous studies^12–14^. The analysis of upper-ocean heat budget reveals that the initialization of subsurface ocean in a CGCM appears to directly improve the predicted zonal heat transport, leading to skillful prediction of the decadal SST variability. Remarkable changes in stratification due to initialization of subsurface ocean temperature and salinity may be responsible for inducing the geostrophic current anomalies afterwards. Since the experiments show that the SST-nudging initialization scheme is not able to precondition the subsurface ocean variability, we note that the subsurface ocean data assimilation considerably helps to improve the predictability of the Southeast Atlantic.

The 3DVAR experiment shows moderately high skills (0.6 ACC) in predicting the decadal SST variability of the Southeast Atlantic, but the prediction skills outside the region do not show much improvement compared to the CTR experiment. Although the choice of the study area is arbitrary to some extent, here we focus on the SEAO region defined previously according to the relevant literature that highlights its potential predictability. However, out of this region, the merits of the subsurface data assimilation appear more questionable and difficult to assess. Since the decadal SST variability in the Southeast Atlantic is large compared to the other regions, the high prediction skills in the Southeast Atlantic may have relations to the large decadal SST variability there. A similar tendency is seen in the other ocean basins using the non-linear statistical prediction^37^, suggesting that there exists an upper limit of prediction skills in the regions where the decadal SST variability is rather small.

Also, the role of atmospheric variability in the decadal SST predictability could not be verified in this study due to lack of long-term observed data. We had only one observed event (2006–2015) to analyze here. Previous studies^12–14^ suggested the role of atmospheric variability based on long-term model simulations in which several events associated with decadal variability are analyzed in a statistical or composite manner. It is expected that more number of observations in the future will help us to properly account for the exact role of the atmospheric variability as reported in those previous modelling studies.

This study provides much evidence for potential roles of subsurface ocean initialization in the decadal SST prediction in the Southeast Atlantic. However, for more realistic application to society, the skillful predictions of surface temperature and rainfall variability over continent and the ocean are indispensable. Both the CTR and 3DVAR experiments represent low skills in predicting SLP anomalies (Fig. S2) which play a key role in determining temperature and rainfall variability. Since the atmospheric circulation in the South Atlantic undergoes decadal variability due to local air-sea interaction^10–14^ and remote influences from the IPO through the atmospheric teleconnection^16^, skillful predictions in the tropical Pacific decadal variability and the South Atlantic SST variability are greatly important. Future work would be required to improve model physics and initialization schemes for the atmosphere (e.g. external forcing such as greenhouse gases), land surface (e.g. soil moisture and snow cover), ocean (e.g. sea-surface height) and sea ice (e.g. sea-ice cover and thickness).

## Methodology

In this study, the decadal climate prediction in the South Atlantic was assessed using historical climate datasets during the satellite period after 1982. The SST data is derived from the Optimum Interpolation SST (OISST) V2^38^ provided by NOAA, whereas the subsurface ocean temperature and salinity are obtained from EN4 quality controlled dataset^39^ provided by Met Office Hadley Centre. The atmospheric data are derived from the ERA-Interim atmospheric reanalysis provided by ECMWF^40^. Here we used monthly mean values with a uniform horizontal resolution of 1° × 1° and calculated monthly anomalies by subtracting monthly climatology from the original product and removing a linear trend related to global warming using a least squared method.

To perform decadal reforecast experiments, we used a CGCM developed under Japan-EU collaboration, called the Scale Interaction Experiment-Frontier Research Center for Global Change 2 (SINTEX-F2) model^41,42^. The SINTEX-F2 model is an upgraded version of SINTEX-F1 model^24,43^. The atmospheric component of the SINTEX-F2 is based on ECHAM5^44^ with 31 levels in the vertical on a T106 Gaussian grid. The ECHAM5 was originally developed at ECMWF and has a parameterization package developed at the Max-Plank Institute for Meteorology. The oceanic component of the SINTEX-F2 is Nucleus for European Modeling of the Ocean (NEMO)^45^, which includes the Louvain-la-Neuve Sea Ice Model 2 (LIM2) sea ice model^46^ and has 0.5° × 0.5° horizontal resolution of ORCA configuration (ORCA05) with 31 levels in the vertical. The atmospheric and oceanic fields are exchanged every 2 hours with no flux correction by means of the Ocean Atmosphere Sea Ice Soil 3 (OASIS3) coupler^47^.

We performed two types of decadal reforecast experiments. One is control (CTR) experiment which is based on the seasonal climate prediction system^28^, but the forecast lead time is extended up to 10 years (Fig. S6a). After 32-yr spun-up with the observed SST climatology since 1950, the model was initialized with SST-nudging scheme every month from 1982 to 2006 in such a way that the model SST is strongly nudged to the observed SST using surface heat flux in the ocean-atmosphere coupled run. The decadal reforecast experiment, then, was performed from Mar 1st of every year from 1982 to 2006, and the model was freely integrated over a 10-year period.

To perform ensemble predictions, we generated 12 different initial conditions in the following manner. For initialization of the model SST, we employed two different SST datasets of the weekly OISSTv2^38^ and the high-resolution daily NOAA OISST analysis^48^. We applied three negative feedback values (−2400, −1200, and −800 W m^−2^ K^−1^) to the surface heat flux in the model, corresponding to 1, 2, and 3-day relaxation time for the 50-m mixed-layer temperature, respectively. The restoring values are set much larger than the value (−40 W m^−2^ K^−1^) proclaimed by the CLIVAR Decadal Climate Variability and Predictability Working Group. This is because our goal here is not the correction of bias, which has by definition longer spatial and temporal scales, but the actual ocean temperature initialization that requires a stronger relaxation within a shorter period. Then, we integrated the model with and without the vertical mixing scheme developed by Sasaki *et al*.^49^. In the scheme, the model takes into account the strong vertical mixing associated with small vertical scale structures (SVSs) within and above the equatorial thermocline. These different initial conditions lead to generating 12 ensemble members for the CTR experiments.

The other experiment, called 3DVAR experiment, is an upgraded version of the CTR experiment involving subsurface ocean initialization (Fig. S6b). Although the SST-nudging scheme is adopted for the initialization phase from 1982, the model’s subsurface ocean temperature and salinity are additionally corrected via 3DVAR data assimilation scheme with 10-day assimilation window at the end of every month^30,31^. For the correction of the subsurface ocean, we used the EN4 subsurface ocean observation data^39^. Same as in the case of the CTR experiment, we performed 10-year lead prediction from Mar 1st of every year from 1982 to 2006 with 12 ensemble members. More detailed information can be referred to Doi *et al*.^32^.

For the above decadal reforecast experiments, the ensemble mean of monthly climatology for the 12 ensemble members was calculated over 10 years (i.e. 120 months) and the model drift (systematic error) was estimated as differences in monthly climatology between the model and the historical datasets. Here a simple averaging technique was used in the calculation of ensemble mean. Monthly anomalies for each ensemble member were calculated by extracting the monthly observed climatology and the model drift from the total values, then by removing the linear trend over the 25-yr experiment period for each lead time.

To investigate physical mechanisms behind the decadal temperature variability in the SINTEX-F2 model, we calculated the temperature tendency anomalies $(\frac{\partial T}{\partial t})^{\prime}$ at each depth averaged over the region of research interest $({Area})$ and their components using the following equation,

1 $$\frac{\rho {c}_{p}}{Area}\iint (\frac{\partial T}{\partial t})^{\prime} dxdy=-\,\frac{\rho {c}_{p}}{Area}\iint \frac{\partial (UT)^{\prime} }{\partial x}dxdy-\frac{\rho {c}_{p}}{Area}\iint \frac{\partial (VT)^{\prime} }{\partial y}dxdy+Residuals$$

where $()^{\prime}$ indicates the monthly anomalies, $\rho$ is the ocean density, and ${c}_{p}$ is the ocean heat capacity. Here we calculated the monthly anomalies by subtracting the monthly climatology and the linear trend over the 25-yr experiment period for each lead time. The first and second terms on the right-hand side represent contributions from convergence anomalies of horizontal $({UT})$ and meridional $({VT})$ heat transport. The last term on the right-hand side is residuals including surface heat flux anomalies, convergence anomalies of vertical heat transport and the remaining heat transport associated with horizontal and vertical mixing. We calculated the residuals by subtracting the first two terms on the right-hand side from the temperature tendency anomaly. For convenience of interpretation, we showed the values without the constant $\rho {c}_{p}$ in Eq. (1).

## Electronic supplementary material

Supplementary Figures
