# Supplementary material for: Role of subsurface ocean in decadal climate predictability over the South Atlantic
Source: Sci Rep. 2018 Jun 4;8:8523. doi: 10.1038/s41598-018-26899-z (PMC5986776; doi:10.1038/s41598-018-26899-z)
Supplement: Supplementary file 1 — Supplementary Figures [file 41598_2018_26899_MOESM1_ESM.pdf]

Supplementary information for

"Role of subsurface ocean in decadal climate predictability over the South Atlantic"

Yushi Morioka[1], Takeshi Doi[1], Andrea Storto[2],

Simona Masina[2,3], Swadhin K. Behera[1]

1: Application Laboratory, JAMSTEC, Yokohama, Japan

2: Fondazione Centro Euro-Mediterraneo sui Cambiamenti Climatici (CMCC), Bologna, Italy

3: Istituto Nazionale di Geofisica e Vulcanologia (INGV), Sezione di Bologna, Bologna, Italy

Corresponding author: Dr. Yushi Morioka

E-mail: morioka@jamstec.go.jp

Supplementary Figure Captions

Figure S1: (a) Annual mean number of subsurface ocean observations in the South Atlantic for each decade during the satellite era. From top to bottom, the in-situ observations using the expendable bathythermograph (XBT), and the conductivity temperature depth (CTD), and the Argo floats are shown, respectively. (b) Longitude-depth sections of annual mean total ocean observations averaged over 50-40ºS of the South Atlantic for each decade. The maps were generated using Grid Analysis and Display System (GrADS) Version 2.1.a3 (http://cola.gmu.edu/grads/downloads.php).

Figure S2: Same as in Fig. 4, but for the detrended SLP anomalies (in hPa). The maps were generated using Grid Analysis and Display System (GrADS) Version 2.1.a3 (http://cola.gmu.edu/grads/downloads.php).

Figure S3: Same as in Fig. 6, but for the detrended subsurface ocean temperature anomalies (in ºC) averaged over 50-40ºS of the South Atlantic from the ORAS4 reanalysis product. Black and gray lines exhibit absolute mixed-layer depth and potential density (C. I. 0.1 Kg m^{-3}), respectively. The maps were generated using Grid Analysis and Display System (GrADS) Version 2.1.a3 (http://cola.gmu.edu/grads/downloads.php).

Figure S4: Same as in Fig. 7, but for the detrended subsurface ocean temperature tendency anomalies (in 10^{-7} ºC s^{-1}) averaged over the SEAO region (black box in Fig. 1c) and their components obtained from the ORAS4 reanalysis product. The maps were generated using Grid Analysis and Display System (GrADS) Version 2.1.a3 (http://cola.gmu.edu/grads/downloads.php).

Figure S5: (a) Year-to-year variations of detrended meridional heat transport anomalies (in 10^{-7} ºC s^{-1}) at the north boundary (40ºS) of the SEAO region (black box in Fig. 1c) from the 3DVAR experiment. For convenience of interpretation, the values are multiplied with minus one. (b) Same as in (a), but at the south boundary (50ºS) of the SEAO region. (c) Same as in (a), but for the detrended meridional current anomalies (in 10^{-2} m s^{-1}) at the north boundary of the SEAO region. (d) Latitude-depth section of detrended subsurface potential density anomalies (in 10^{-1} Kg m^{-3}) at the north boundary of the SEAO region. Black and gray lines exhibit absolute mixed-layer depth and potential density (C. I. 0.1 Kg m^{-3}), respectively. The maps were generated using Grid Analysis and Display System (GrADS) Version 2.1.a3 (http://cola.gmu.edu/grads/downloads.php).

Figure S6: Schematic diagrams of (a) CTR and (b) 3DVAR decadal reforecast experiments. In the CTR experiment, the decadal reforecast run initiates from March 1st of every year from 1982 to 2006 after the model SST is initialized using the SST-nudging scheme. On the other hand, in the 3DVAR experiment, the model temperature and salinity in the subsurface ocean are additionally initialized using the 3DVAR assimilation scheme every month at the end of the SST initialization. Then, the decadal reforecast run initiates from March 1st of every year from 1982 to 2006.

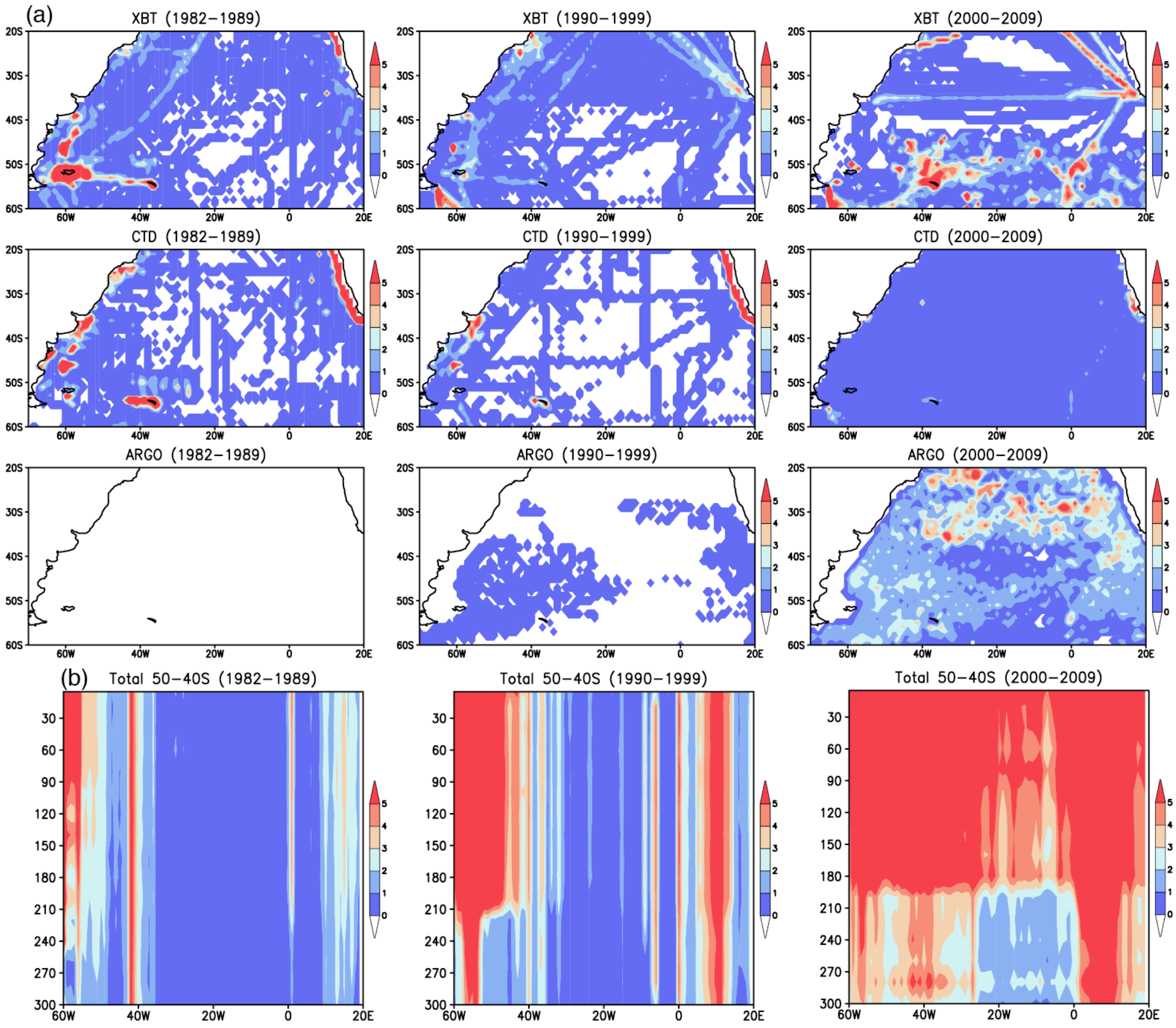

Figure S1: (a) Annual mean number of subsurface ocean observations in the South Atlantic for each decade during the satellite era. From top to bottom, the in-situ observations using the expendable bathythermograph (XBT), and the conductivity temperature depth (CTD), and the Argo floats are shown, respectively. (b) Longitude-depth sections of annual mean total ocean observations averaged over 50-40°S of the South Atlantic for each decade. The maps were generated using Grid Analysis and Display System (GrADS) Version 2.1.a3 (http://cola.gmu.edu/grads/downloads.php).

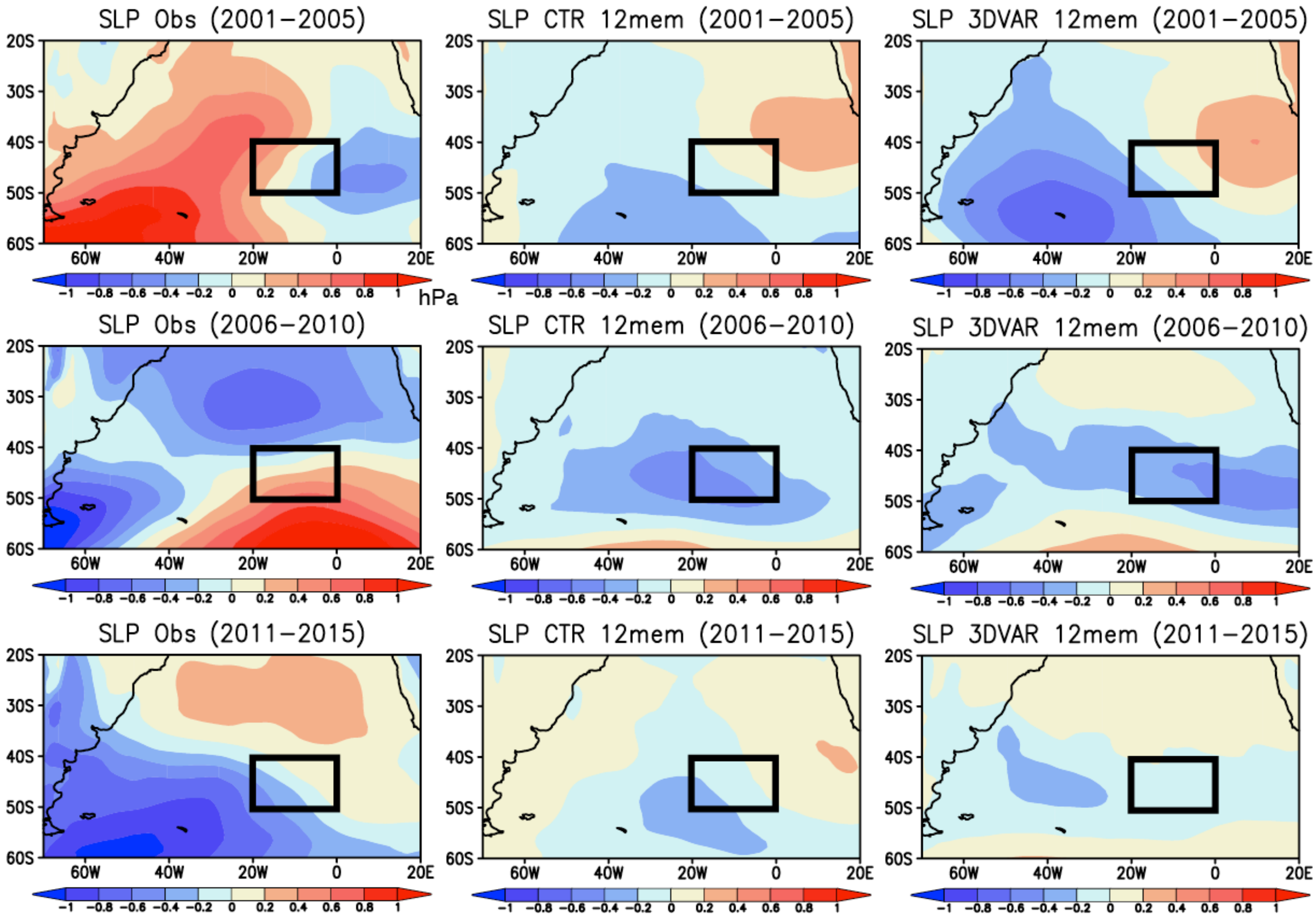

Figure S2: Same as in Fig. 4, but for the detrended SLP anomalies (in hPa). The maps were generated using Grid Analysis and Display System (GrADS) Version 2.1.a3 (http://cola.gmu.edu/grads/downloads.php).

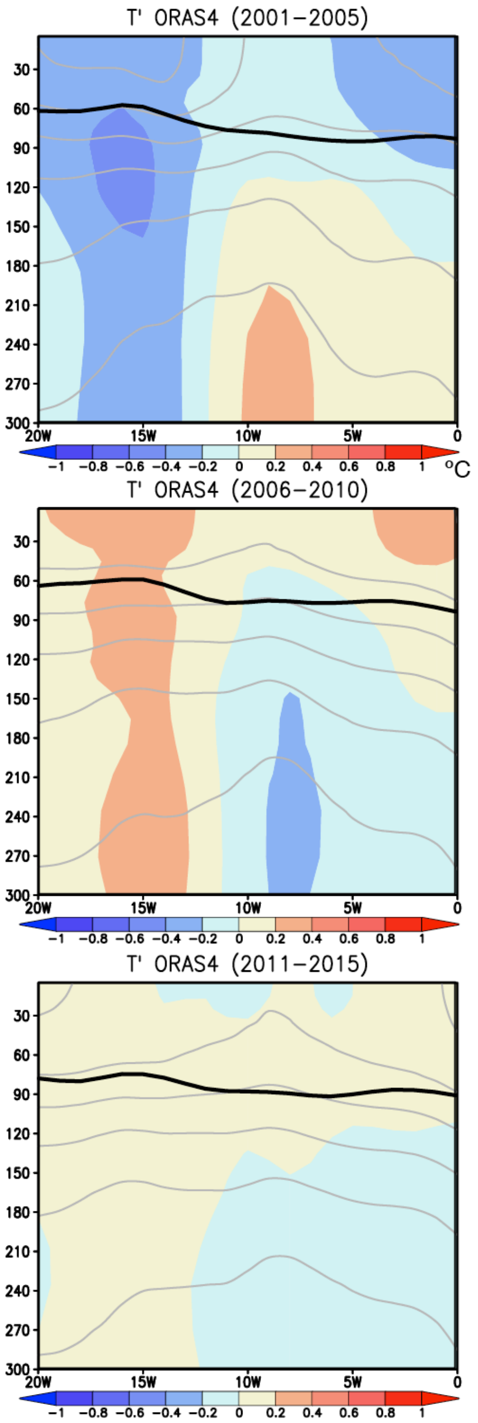

Figure S3: Same as in Fig. 6, but for the detrended subsurface ocean temperature anomalies (in ºC) averaged over 50-40ºS of the South Atlantic from the ORAS4 reanalysis product. Black and gray lines exhibit absolute mixed-layer depth and potential density (C. I. 0.1 Kg m^{-3}), respectively. The maps were generated using Grid Analysis and Display System (GrADS) Version 2.1.a3 (http://cola.gmu.edu/grads/downloads.php).

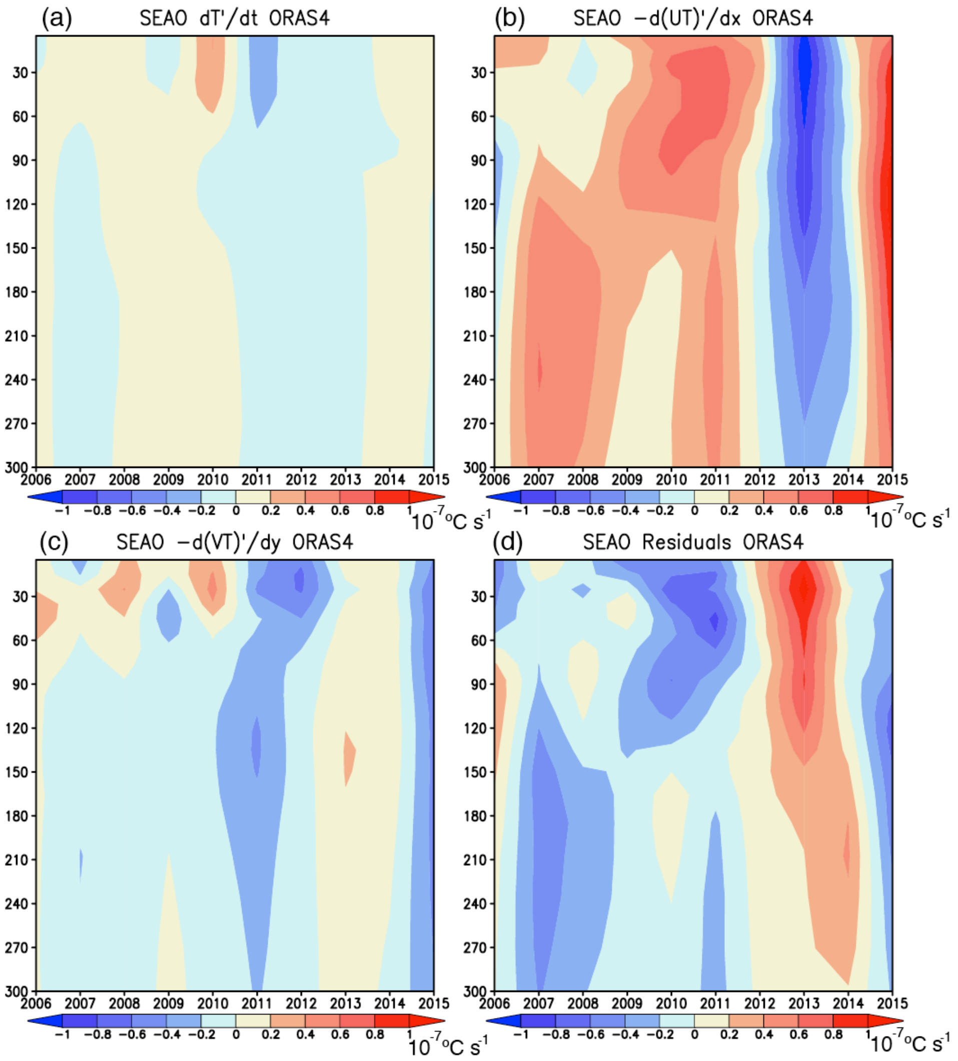

Figure S4: Same as in Fig. 7, but for the detrended subsurface ocean temperature tendency anomalies (in 10^{-7} ºC s^{-1}) averaged over the SEAO region (black box in Fig. 1c) and their components obtained from the ORAS4 reanalysis product. The maps were generated using Grid Analysis and Display System (GrADS) Version 2.1.a3 (http://cola.gmu.edu/grads/downloads.php).

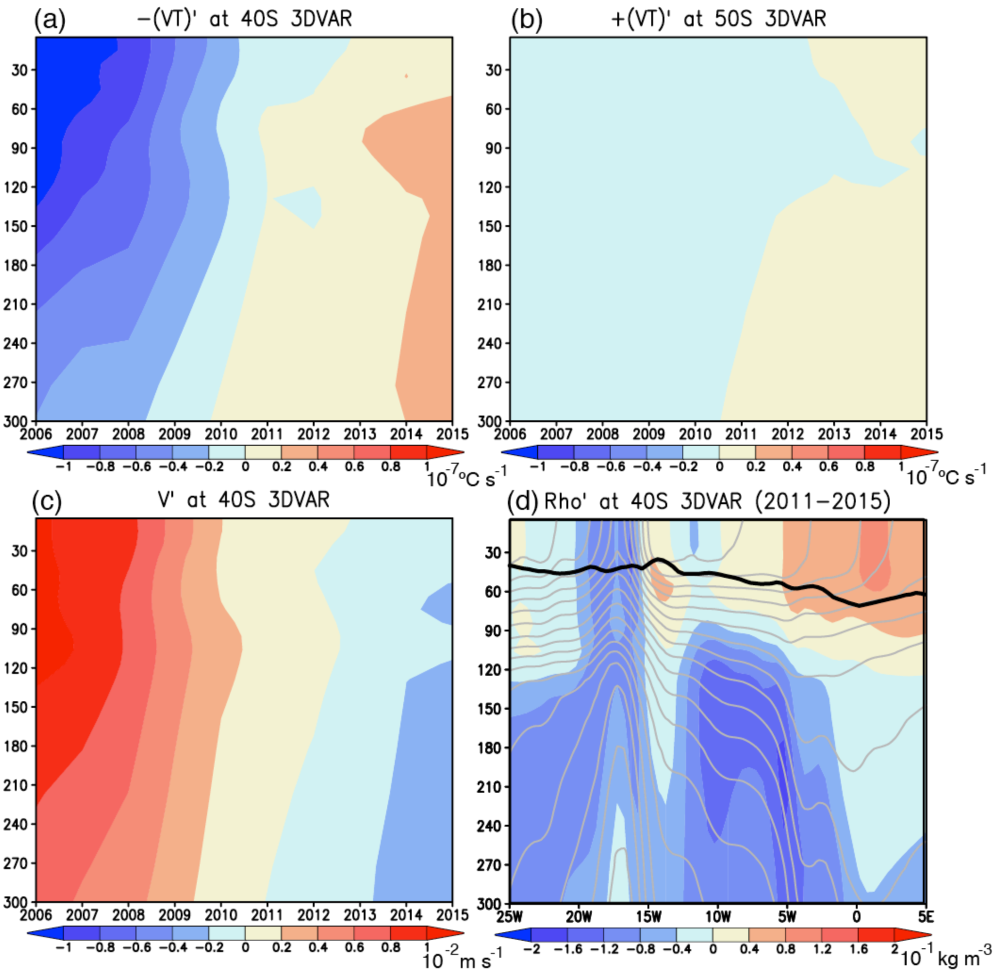

Figure S5: (a) Year-to-year variations of detrended meridional heat transport anomalies (in 10^{-7} ºC s^{-1}) at the north boundary (40ºS) of the SEAO region (black box in Fig. 1c) from the 3DVAR experiment. For convenience of interpretation, the values are multiplied with minus one. (b) Same as in (a), but at the south boundary (50ºS) of the SEAO region. (c) Same as in (a), but for the detrended meridional current anomalies (in 10^{-2} m s^{-1}) at the north boundary of the SEAO region. (d) Latitude-depth section of detrended subsurface potential density anomalies (in 10^{-1} Kg m^{-3}) at the north boundary of the SEAO region. Black and gray lines exhibit absolute mixed-layer depth and potential density (C. I. 0.1 Kg m^{-3}), respectively. The maps were generated using Grid Analysis and Display System (GrADS) Version 2.1.a3 (http://cola.gmu.edu/grads/downloads.php).

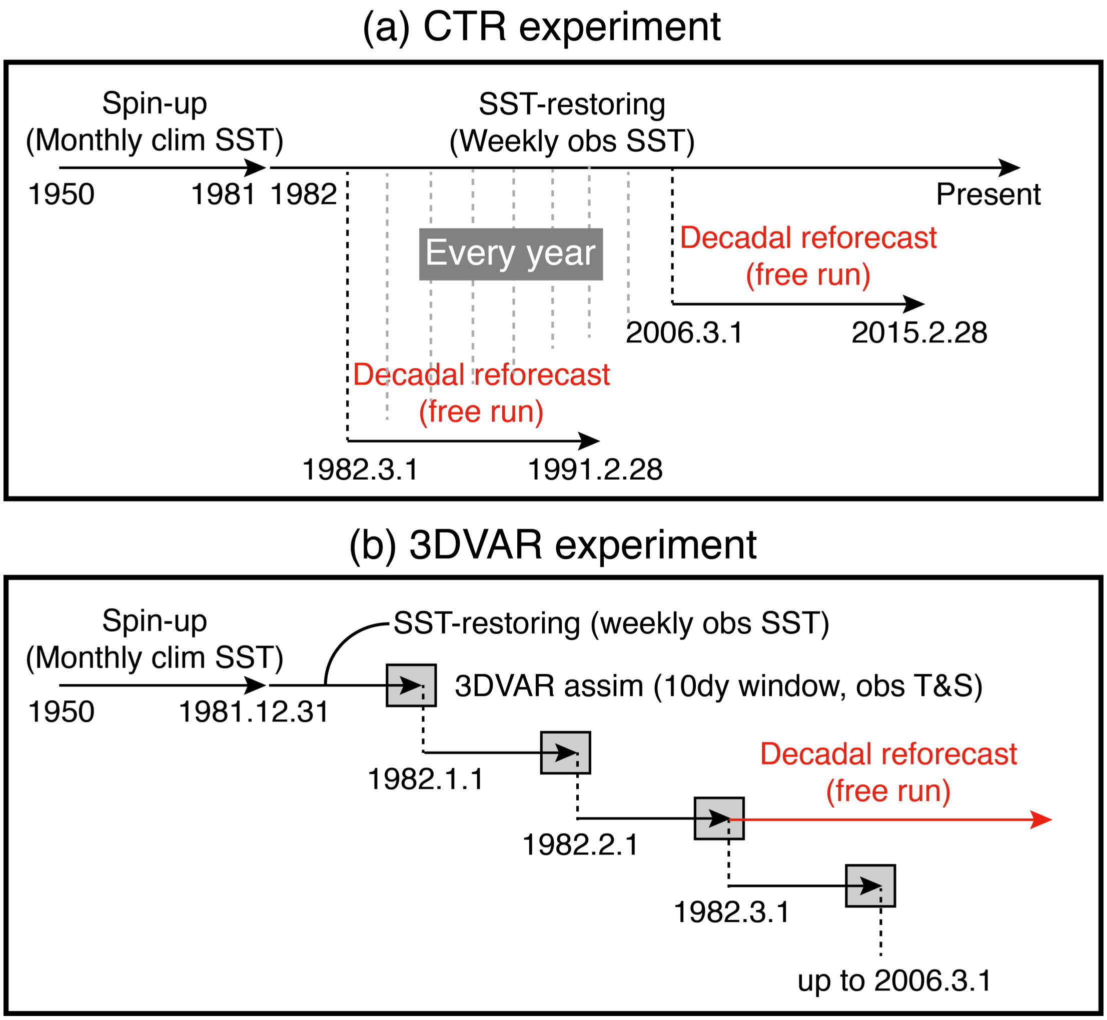

Figure S6: Schematic diagrams of (a) CTR and (b) 3DVAR decadal reforecast experiments. In the CTR experiment, the decadal reforecast run initiates from March 1st of every year from 1982 to 2006 after the model SST is initialized using the SST-nudging scheme. On the other hand, in the 3DVAR experiment, the model temperature and salinity in the subsurface ocean are additionally initialized using the 3DVAR assimilation scheme every month at the end of the SST initialization. Then, the decadal reforecast run initiates from March 1st of every year from 1982 to 2006.
